# Sexual dysfunction and marital satisfaction among the chemically injured veterans

**DOI:** 10.4103/0970-1591.36710

**Published:** 2007

**Authors:** Khodabakhsh Ahmadi, Hossein Ranjebar-Shayan, Fateme Raiisi

**Affiliations:** The Behavioral Sciences Research Center, Baqiyatallah Medical Sciences University, Tehran, 14548, I.R, Iran

**Keywords:** Chemically injured veterans, marital satisfaction, sexual dysfunction

## Abstract

**Introduction:**

Researches show that chronic illnesses may affect marital adjustment and sexual function. Therefore, the purpose of this research was to recognize marital satisfaction, sexual dysfunction and demographic factors among Chemically Injured Veterans (CIV).

**Materials and Methods:**

In this descriptive research, we selected and studied 185 cases of CIVs referred to Tehran's hospitals. Data gathering tools were Enrich Marital Satisfaction Scale and structured interview. The items of interview included 28 questions about sexual dysfunction according to DSM-IV-r. The statistical methods were T-test, ANOVA and Correlation.

**Results:**

The results show that 45.5%, i.e. nearly half of the CIV subjects were dissatisfied with their marriage and marital relationship and the dissatisfaction level in 11% was very high. Other results show that 65/4% of veterans with chemical injuries suffered from a kind of sexual dysfunction. The most common dysfunctions were: erectile problem and libido reduction by 49.2% and 48.6% respectively. Also, results show that there was a relation between demographic factors and sexual dysfunction in CIVs.

**Discussion:**

As far as the results show, sexual libido reduction rate in CIVs is like that in chronic obstructive pulmonary disease patients. Therefore, sex therapy, psychotherapy and couple therapy must be a component of the treatment of CIVs.

Studies show that physical disabilities and chronic diseases can impact one's sexual functioning in different aspects. They can also affect marital relationship and satisfaction from a sexual partner. In a study, men 50 to 70 years old were surveyed for impotence, the effect of aging and its treatment conditions for a period of five years. Impotence was studied with two questions about erection initiation and its maintenance during coitus.

Results of the study revealed that during five years, the rate of mild erectile dysfunction increased 40% from 127 per 1000, average erectile dysfunction increased 80% from 41 per 1000 and severe erectile dysfunction increased 190% from 18 per 1000. Besides, the rate of erectile dysfunction was higher in men with diabetes (2.4 % for mild dysfunction, 2.6% for moderate dysfunction and 3.4% for complete dysfunction). Also hypertension, cardiac diseases, arthritis, pulmonary diseases and neurovascular disorders had no or little effect on erectile dysfunction. The survey showed that erectile dysfunction is a common disorder between the ages 50 to 70, on which diabetes has high impact while other diseases have little effect.[1]

Studying sexual functioning of alcoholics showed that one-third of them suffer from erectile dysfunction, loss of libido, premature or delayed ejaculation. A follow-up study was done nine months after the end of treatment. No significant differences in the prevalence of sexual dysfunction were found between the two points of measurement. All patients had normal levels of plasma testosterone at the beginning and end of inpatient treatment. These findings suggest psychological causes for the sexual problems and a need for therapeutic intervention. Furthermore, follow-up results indicate that the treatment group who received behavioral treatment, showed significantly less sexual dysfunction than an untreated control group.[2] Erectile dysfunction is common among men with respiratory dysfunction and hypoxia. Different studies show the relationship between oxygen absorption rate and erectile dysfunction. In a study, five out of 12 patients who had received oxygen for one month had their sexual function restored. As a result, both their arterial PO_2_ and their serum testosterone have increased. This study showed that oxygen should be administered for a long time.[3]

In another study 20 men 46 to 69 years old, who suffered from COPD, were investigated. Seven subjects had ceased sexual activity concomitant with worsening of their pulmonary symptoms; six because of erectile impotence and one due to dyspnea. Frequency of intercourse for the remaining 13 was 16% of pre-lung disease levels and libido was decreased to 25% of pre-morbid levels. Six subjects had organogenic erectile impotence (OEI), but none showed signs of peripheral vascular disease. Four subjects had occult diabetes mellitus and one had evidence of an androgen deficit. The correlation coefficient for rank by sexual dysfunction vs. pulmonary impairment and age was 0.66 (*P* less than 0.005) and 0.24 (*P* greater than 0.05), respectively. Subjects with OEI tended to have the highest T-scores on the hypochondriasis, depression and hysteria scales of the MMPI [Minnesota Multiphase Personality Inventor] test.[4] In another study, 49 male COPD patients with chronic respiratory failure on long-term oxygen therapy were studied. Thirty-three patients (67.3%) showed some type of sexual problem (lack of desire and/or impotence). Sixteen wives (33%) answered affirmatively to the question about whether changes at a communicative level as a consequence of the patients’ illness had occurred. The wives were significantly less satisfied with the relationship than the patients, which were related to communication problems. A factor which influences the perception of such problems in a very important way is the degree of affection in the relationship between the couples.[5]

Another study on sexual status and erectile dysfunction (ED) in outpatients with COPD showed nearly half of the patients (49%) had no comorbid disease for ED and the most common comorbid disease was hypertension (34%). According to the erectile function domain of IIEF, 75.5% of patients were found to have ED with varying degrees (severe 28.3%, moderate 11.3%, mild to moderate 15.1% and mild 20.8%). Mean scores of all IIEF domains except sexual desire decreased with the increasing disease severity. A correlation was determined between severity and physical restrictions of COPD and ED severity. It was concluded that the limitation of physical activity due to COPD also diminishes the sexual function of patients. This point must be kept in mind in the evaluation of patients with COPD.[6] Data suggest that sexual dysfunction worsens as lung disease worsens and that COPD may be associated with male impotence in the absence of other commonly known causes.[4]

It can be assumed that there is a relation between somatic diseases and male sexual dysfunction. Based on this, we can say that somatic diseases can affect sexual functioning, which is related to both somatic and psychological factors. An important point is that the possible somatic cause of sexual dysfunction cannot disprove psychological factors and that organic sexual dysfunction is not solely somatic. It is then concluded that one of the consequences of chronic diseases is lack of sexual function.[7] That is why we aim to study the rate of sexual dysfunction in the chemically wounded-in-action (WIA). It is well established that mustard gas causes the same symptoms and signs as COPD.

Numerous interactions between the biopsychosocial stressors and functioning (including coping strategies and patterns) within and between marital partners dealing with dyspnea and the psychosocial consequences of COPD are described. The review is expanded to encompass the reciprocal influences between marriage and chronic illness. A case is made for assessing and addressing health needs using a systems perspective which takes into account the realities of both marital partners as they manage their lives together. Implications for approaching multidisciplinary healthcare within the interpersonal context of marriage are suggested.[8] Therefore, the chronic disease may effects on the marital adjustment and health of the couples.[9] Marital adjustment has been associated with morbidity and mortality across various chronic diseases but has been largely ignored among patients with COPD. The studies show that marital adjustment is associated with both psychological wellbeing and physical functioning among patients with COPD.[10] There is evidence suggesting an association between marital functioning and symptoms of COPD. For example, disability resulting from dyspnea can lead to loss of social, vocational and recreational activities, which, in turn, can result in impaired marital adjustment because couples are no longer able to engage in the activities they once enjoyed.[11] Likewise, cognitive impairment associated with COPD may disrupt mood and daily functioning in the patient and partner[12,13] and which, in turn, may have a negative effect on the partner[14] and on marital adjustment. Disability resulting from the psychological and physical effects of COPD also may lead to sexual inactivity in patients with COPD.[15] For example, chronic coughing, urinary incontinence during coughing, excessive phlegm production and concerns about physical changes in appearance (e.g. excessive weight loss) may have a negative impact on sexual functioning. It has been reported that up to 74% of patients who have COPD experience decline in sexual functioning.[16] Impotence or suspended intercourse caused by dyspnea has been estimated to occur in 35% of men with COPD.[17] The wives of COPD patients reported higher subjective stress and lower life satisfaction than the wives whose husbands did not have a chronic illness. The COPD wives assumed more new roles and responsibilities, relinquished more social activities, rated their health lower and reported less frequent marital relations.[18]

## MATERIALS AND METHODS

This descriptive study enrolled all CIVs of the Iran-Iraq war who were admitted to clinics in Tehran in the spring and summer of 2006. The sample size of 185 included both chemical and non-chemical veterans. The sample was calculated by considering X=5% and *P*=40% (Kaplan reference). Inclusion criteria were being married and admission to Baqiyatallah University of Medical Sciences Chemical Injuries Clinic, Baqiyatallah Hospital, Sasan Hospital, Khatamolanbia Hospital and other related clinics.

For data gathering, we used ENRICH marital satisfaction scale and sexual dysfunction checklist according to DSM-IV-r.[19] The ENRICH main test copy includes 115 questions. This form was first used for the description of dynamism of marriage and then as an equipment for diagnosis of couples who were seeking marriage counseling. The questionnaire validity index in the clinical affairs was between 0.85 and 0.95.[20,21] This questionnaire includes subscales such as: personality issues, marital communication, conflict resolution, financial problems, leisure activities, sexual relationship, parenting, family and friends and religiosity.[22] Under study sexual dysfunctions in this research include: lack of libido, lack of sexual desire, decrease of arousal, premature ejaculation, hatred of sexual intercourse, impotence, loss of orgasm, hatred of intercourse, erectile dysfunction, ejaculation delay, premature ejaculation, painful ejaculation, dry ejaculation and masturbation. Therefore, a 28-question questionnaire was designed and filled out in an interview. It had two parts: one for demographic data and another for sexual dysfunction based on DSM-IV-r,[19] which the researcher changed into yes/no questions. Then, questions were integrated and sent to psychiatrists, urologists and psychologists for comments and modifications. After modifications, the questionnaire was approved by the ethics committee of the Baqiyatallah University of Medical Sciences. The questionnaire was filled out by the specialist during the interview with the patient. The questionnaire was validated by Kronbach's alpha of 0.79 and Split-half of 0.82. Data were analyzed using SPSS, t-test, one-way variance and correlation coefficient.

## RESULTS

Out of 185 subjects [Table 1], 38.6% did not have high-school diploma, 24.2% had high-school diploma and 27.2% had higher education. Their mean age was 43.4±4.2 and all were married. Out of 185 subjects, 56.5% had civilian occupations while 32.1% were servicemen and 11.4% were unemployed. All subjects were hit by chemical warfare during the Iraq-Iran war, so they had at least 17 years and at most 25 years of injury with a mean of 21.3±2.2. The severity of injury was at least 15% and at most 70%, with a mean of 23.3±11.7%. They enjoyed a married life for at least five years and at the most 40 years, with a mean of 20.1±5.5.

**Table 1:** Demographic data of the subjects

Variable	Minimum	Maximum	Mean	SD
Age	37 years	57 years	43.4	4.2
Duration of chemical injury	17 years	25 years	21.3	2.2
Severity of injury	15%	70%	23.3	11.7
Duration of married life	5 years	40 years	20.1	5.5
Level of education	Under high-school diploma	High-school diploma		Higher education
	Frequency:72	Frequency: 63		Frequency: 50
	Percentage:38.6	Percentage: 43.1		Percentage:27.3
Occupation	Civilian	Servicemen		Unemployed
	Frequency: 105	Frequency: 59		Frequency: 21
	Percentage:56.5	Percentage: 32.1		Percentage: 11.4

The results show that 31.9% of the subjects were dissatisfied with their marriage and marital relationship and the dissatisfaction level in 11.3% was very high. The subjects’ marital satisfaction was borderline in 62.4% of cases. Only 5.7% were happy with their marital relationships. Perfect marital satisfaction was not seen at all. The presented figures show that marital satisfaction in veterans has a little positive skew in respect to their relations and their average satisfaction is lower than that in the general population [Table 2].

**Table 2:** Marital satisfaction among chemically injured veterans

Marital satisfaction status	frequency	Percentage
Strong dissatisfaction	21	11.3
Dissatisfaction	38	20.6
Borderline	115	62.4
Strong satisfaction	11	5.7
Extreme satisfaction	0	0

In case of sexual dysfunction, 65.4% of all subjects suffered from a kind of sexual dysfunction and the remaining 34.6% had no such problems. Of those with sexual dysfunction, only 25.6% sought medical advice for their problem. Of all subjects, only 3.8% abused drugs [Table 3].

**Table 3:** Rate of sexual dysfunction, history of seeking medical advice and drug abuse among chemically injured veterans

Item	Status	Frequency	Percentage
Sexual dysfunction	Positive	121	65.4
	Negative	64	34.6
Hx of seeking medical advice	Positive	31	25.6
	Negative	90	74.4
Hx of drug abuse	Positive	7	3.8
	Negative	178	96.2

The rate of impotence and lack of sexual desire were the most common problems among CIVs (49.2% and 48.6%, respectively). Other disorders in decreasing order are delayed ejaculation (22.2%), inability to have intercourse (17.8%), premature ejaculation (17.8%) orgasmic anhedonia (14.1%) and lack of sexual fantasy (13%) [Table 4].

**Table 4:** Kinds and rates of sexual dysfunction among CIVs

Kind of disorder	Frequency	Percentage
Impotence	91	49.2
Lack of sexual desire	90	48.6
Delayed ejaculation	54	29.2
Lack of libido	41	22.2
Inability to have intercourse	33	17.8
Premature ejaculation	33	17.8
Orgasmic anhedonia	26	14.1
Lack of sexual fantasy	24	13

Since some of the subjects had more than one disorder, the sum of rates is more than 100%.		

Studying the correlation of demographic factors with sexual dysfunction among CIVs revealed an association with level of education, occupation and history of seeking medical advice (P≤0.01) and severity of injury (P≤0.05). On the other hand, history of mental disorders, history of drug abuse, age, duration of chemical injury and duration of marriage showed no relation with sexual dysfunction (P≤0.05) [Tables 5 and 6].

**Table 5:** Association between demographic factors and sexual dysfunction among chemically injured veterans using χ^2^ test

Variable	Level	Rate of subjects with sexual dysfunction	Rate of subjects without sexual dysfunction	χ^2^	*P*	Difference
Level of education	Under high-school	78.9	21.1	8.45	0.01	1>2,3
	High-school anddiploma	58.7	41.3		**	
	Higher education	57.1	42.9			
Occupation	Servicemen	48.3	51.7	16.49	0.0003	3>2>1
	Civilian	69.2	30.8		**	
	Unemployed	95.2	4.8			
Hx of seeking	Positive	96.8	3.2	15.92	0.0001	1>2
medical advice	Negative	59.5	40.5		**	
Hx of mental	Positive	71.8	28.2	2.19	0.14	-
disorders	Negative	61.3	38.7		-	
Hx of drug abuse	Positive	85.7	14.3	1.29	0.26	-
	Negative	65	35		-	

- No significance

* *P*<0.05

** *P*<0.01

**Table 6:** Association of demographic factors with sexual dysfunction among chemically injured veterans using t-test

Variable	Groups	Mean	SD	t	*P*	Difference
Age	With sexual dysfunction	43.4	4.6	0.34	0.73	-
	Without sexual dysfunction	43.2	3.2		-	
Duration of chemical injury	With sexual dysfunction	21.1	2.3	-1.22	0.22	-
	Without sexual dysfunction	21.6	2.1		-	
Severity of injury	With sexual dysfunction	24.5	12.3	1.94	0.05	1>2
	Without sexual dysfunction	20.6	10		*	
Duration of marriage	With sexual dysfunction	19.9	5.9	-1.14	0.26	-
	Without sexual dysfunction	20.8	3.9		-	

- No significance

* *P*<0.05

** *P*<0.01

Accordingly, it is clear that CIVs who do not hold high-school diploma suffer from sexual dysfunction more than others, unemployed ones more than others, civilians more than servicemen and those who did not seek medical advice more than those who did. Also, the higher the severity of injury, the higher the rate of sexual dysfunction.

## DISCUSSION

Veterans afflicted with chemical injuries will confront gradual death. There have precocious death. On the one hand, the suffering of chemicals and on the other hand the idea of precocious death makes the veteran and his family worried and stressed. Other problems such as treatment follow-up, role change, fear of disease transmission and nursing, which are mostly on the wives’ shoulders, besides limitations in family and social relations aggravate the situation.

Therefore, stress symptoms among CIVs may be more. Perhaps these symptoms present initially in the family, which increases the stress level within the family. The first person affected by this is the veteran's wife. Therefore, marital maladjustment is more at stake than other familial aspects in the families of such veterans. According to previous researches[10] and as the present study suggests, the rate of marital dissatisfaction in CIVs is 31.9% and 62.4% of CIVs do not enjoy marital life and experience conflicts, for the removal of which, both partners do their best. The present study confirms the finding of Cannon, Cavanaugh and others, which states that more COPDs and chronic diseases have marital maladjustment. This study showed that 45.5% of veterans had marital maladjustment and 11% of them had extremely severe maladjustment.[6] There is, however, a difference in Iranian veterans in terms of cultural background; they mostly get married for spiritual reasons. Due to the spiritual atmosphere of Iran and the fact that veterans are considered ‘living martyrs’, some girls voluntarily get married to them and are committed to their marriage. Bearing difficulties is also considered to be rewarded in the other world and a way to reach paradise. Based on these results, the veteran's spouse can and some of those are considered his nurse, as well and earns a salary for that, but there are no regular consultation services and psychotherapy for them in stressful conditions. Marriage counseling is essential for the first group (the group which has marital maladjustment comprises 31.9% of those veterans). The second group also needs counseling to help prevent further family problems (those whose marital relation is sometimes good and sometimes bad are considered borderline and comprise 62.4% of CIVs).

There is a mutual relationship between somatic diseases and male sexual dysfunction. Accordingly, somatic diseases can affect sexual functioning, which is related to both somatic and psychological factors.[7] Based on the findings of research, 65.4% of CIVs have a kind of sexual dysfunction. The mean age of the subjects was 43.4±4.2 years. We also know that erectile disorders increase after the age of 50.[1] Therefore, it is predictable that the rate and severity of impotence and sexual dysfunction will increase in CIVs. That's why it seems necessary to add sexual functioning improvement to their treatment regimen.

Since sexual dysfunction has somatic and psychological bases[7] and it is also affected by marital relationship, personality characteristics and psychological disorders vice versa, we can conclude that the main part of treatment for sexual dysfunction of CIVs is psychological treatment. Studies show that behavior therapy[2] and cognitive-behavior therapy[23] and couple therapy can improve sexual functioning.

Eight kinds of sexual dysfunction were studied here, two of them were the most common among CIVs, that is, impotence by the rate of 49.2% and lack of sexual desire by the rate of 48.6%. As seen in other studies,[5] impotence is more prevalent in patients with COPD and the rate is about the same as we found in this study. Therefore, cultural differences and special conditions of CIVs do not affect the prevalence of impotence among them. We can conclude that it is the consequence of the disorders caused by chemicals.

Furthermore, 48.6% of the subjects lack sexual desire, which can be the result of impotence. In addition, it can hurt sexual relations and personality issues.[5]

Among demographic factors, level of education, occupation, history of seeking medical advice for sexual disorders and the rate of chemical injury affect sexual dysfunction. It is evident that those subjects with lower levels of education have more sexual dysfunction. It can be assumed that they do not have knowledge or enough knowledge of sexual relationship and the ways to improve it. This hypothesis can be tested in another study.

We also found that unemployed subjects more than others and civilian subjects more than servicemen were affected by sexual dysfunction.

Another finding of this study was that the severity of chemical injury affects sexual dysfunction. We can conclude that there is an association between sexual dysfunction and severity of chemical injury. Besides, sexual dysfunction is not caused by occupation, while it is caused by chemical injury those with severe injuries are either unemployed or disabled.

It should be mentioned that the data of this study describe and demonstrate the CIVs group. These results can only generalize the ability for this group. The reader can compare these results with other related articles which have evaluated other groups. For example, the research with stressfully groups had shown that: sexual dysfunction on Veteran's PTSD was 89.9% and marital dissatisfaction in this group was 44.5%.[24] In this manner, the marital adjustment on Widows of the Killed-in-Action vs. Other Widows were meaningful upper (39.3% versus 22.2%).[25]

In conclusion, we know that sexual dysfunction, especially impotence and lack of sexual desire are prevalent among CIVs, which is nearly the same as that in COPD patients. It is expected to increase with increase in age. Therefore we recommend psychotherapy and couple therapy to be included in the CIVs’ treatment regimen.
